# Systemic Lupus Erythematosus Presenting Primarily With End-Stage Renal Disease in Men: A Case Report

**DOI:** 10.7759/cureus.91216

**Published:** 2025-08-29

**Authors:** Zelun Li, Kaiying Yang

**Affiliations:** 1 Department of Critical Care Medicine, Panyu Hexian Memorial Hospital of Guangzhou, Guangzhou, CHN

**Keywords:** case report, infection, multisystem damage, renal insufficiency, systemic lupus erythematosus

## Abstract

This case report describes a diagnostically challenging presentation of systemic lupus erythematosus (SLE) in a 37-year-old male patient with a five-year history of isolated chronic kidney disease (CKD) requiring hemodialysis, who acutely developed progressive multi-organ failure. Initial evaluation revealed severe pancytopenia, hypoalbuminemia, elevated cardiac biomarkers, and multi-cavity effusions. Autoimmune serology confirmed the diagnosis of SLE with characteristic autoantibodies. Critical complications included refractory septic shock, extensive coronary artery disease, splenic infarction, cerebral hemorrhage, and terminal multi-organ failure. Despite multidisciplinary intervention, rapid clinical deterioration resulted in a fatal outcome. This case highlights that isolated CKD can obscure SLE onset in men, emphasizing the need for early serological screening in unexplained multisystem deterioration, even in the absence of classic lupus features, to prevent diagnostic delays and fatal complications. To our knowledge, this is the first report of SLE in a male patient presenting as isolated CKD for five years before fatal multi-organ failure, highlighting gender-specific diagnostic challenges.

## Introduction

Systemic lupus erythematosus (SLE) is a chronic autoimmune disease characterized by multi-organ involvement and heterogeneous clinical manifestations. SLE predominantly affects women of childbearing age, and male patients account for only 10%-20% of cases, often presenting with more severe renal and hematological complications [1-3]. This gender disparity may contribute to delayed diagnosis in men due to lower clinical suspicion [2,3].

A notable challenge in SLE management is its variable initial presentation. Although renal involvement (lupus nephritis) is common during disease progression, isolated renal dysfunction as the inaugural symptom is rare and easily misdiagnosed as primary chronic kidney disease (CKD). Studies suggest that <5% of SLE cases initially manifest with significant renal impairment without classic features such as cutaneous or articular symptoms [4]. This atypical presentation poses diagnostic dilemmas, particularly in patients with pre-existing CKD, where overlapping etiologies (e.g., hypertension and chronic glomerulonephritis) may obscure underlying autoimmune pathology [4,5].

This case report describes a 37-year-old man with a five-year history of CKD who developed fatal multi-organ failure ultimately attributed to SLE. The absence of typical lupus signs (e.g., malar rash and photosensitivity) and the dominance of renal dysfunction masked the autoimmune etiology until advanced stages, underscoring the importance of considering SLE in men with unexplained progressive multisystemic deterioration [1,6]. Furthermore, this case highlights the critical need for early immunological evaluation in patients with chronic renal disease, even in the absence of classic autoimmune features, to mitigate life-threatening complications [4,5].

## Case presentation

This case presented with renal insufficiency as the initial symptom and was ultimately diagnosed as SLE. A 37-year-old man was admitted to the nephrology department on January 16, 2023, due to "shortness of breath and fatigue for one week." Three weeks prior to admission, he experienced coughing, sputum production, chest tightness, shortness of breath, and recurrent hematochezia, along with poor appetite, hypoglycemia, and occasional seizures that would resolve spontaneously; one week prior, his shortness of breath worsened after activity. He denied fever, chest tightness, chest pain, and urinary tract irritation symptoms. His medical history included chronic renal insufficiency for five years, with regular hemodialysis three times a week, and a four-year history of hypertension, managed with sustained-release nifedipine and valsartan, keeping his blood pressure between 120 and 140/80 and 100 mmHg. He had no history of smoking or alcohol use. He denied any history of allergies, trauma, surgeries, or infectious diseases; he also denied any family history related to the current illness.

Admission examination

His temperature was 36.7°C, heart rate was 113 beats/minute, respiration rate was 22 breaths/minute, and blood pressure was 141/106 mmHg, with an alert mental state, and anemic and chronic disease appearance. He was brought in a wheelchair into the ward, with slightly labored breathing, coarse breath sounds in both lungs, and bilateral moist rales. His heart rhythm was regular, with no pathological murmurs heard in any valves. The abdomen shows no tenderness or rebound tenderness, no percussion pain in both kidney areas, and no edema in both lower limbs.

Laboratory findings

His hematological and biochemical findings are presented in Table 1, and his autoimmune serology findings are presented in Table 2.

**Table 1:** Hematological and Biochemical Findings WBC: white blood cell count, RBC: red blood cell count, PLT: platelet count, Hb: hemoglobin, BNP: brain natriuretic peptide, CRP: C-reactive protein, PCT: procalcitonin, LAC: lactic acid, Cr: creatinine, BUN: blood urea nitrogen, CK: creatine kinase, CK-MB: creatine kinase-MB, TSH: thyroid-stimulating hormone

Test	Result (Date)	Reference Range	Units
WBC	3.48 (January 16)	4.0-10.0	×10⁹/L
RBC	2.87 (January 16)	4.3-5.8	×10¹²/L
PLT	72 (January 16)	100-300	×10⁹/L
Hb	88 (January 16)	130-175	g/L
Albumin	28.7 (January 16)	39.7-49.4	g/L
BNP	1267.96 (January 16)	<100	pg/mL
CRP	12.4 (January 16)	<10	mg/L
PCT	5.14 (January 16)	<0.05	ng/mL
LAC	1.08 (January 16)	0.5-2.44	mmol/L
Cr	1429 (January 16)	31-93	μmol/L
BUN	38.1 (January 16)	2.86-8.2	mmol/L
CK	147 (January 16)	24-190	U/L
CK-MB	14 (January 16)	0-25	U/L
High-sensitivity troponin	177.1 (January 16)	0-19	ng/L
TSH	6.73 (January 17)	0.27-4.2	mIU/L

**Table 2:** Autoimmune Serology Findings ds-DNA: dsDNA antibody, ANA: antinuclear antibody, AnuA: anti-nucleosome antibody, ACA: anti-cardiolipin antibody, ANCA-PR3: anti-PR3 antibody, ANCA-MPO: anti-MPO antibody, DNP-Ab: deoxyribonucleoprotein antibody

Test	Result (Date)	Reference Range
Anti-ds-DNA	1:40 (February 15)	Negative (<1:10)
ANA	1:320 (February 15)	Negative (<1:80)
AnuA	Positive (February 15)	Negative
ACA	Positive (February 15)	Negative
ANCA-PR3	Negative (February 15)	Negative
ANCA-MPO	Negative (February 15)	Negative
Anti-DNP-Ab	Negative (February 15)	Negative
Anti-Jo-1 antibody	Negative (February 15)	Negative
Anti-Scl-70 antibody	Negative (February 15)	Negative
Anti-SSB antibody	Negative (February 15)	Negative
Anti-SM antibody	Negative (February 15)	Negative
Anti-SSA antibody	Negative (February 15)	Negative
Anti-U1-snRNP antibody	Negative (February 15)	Negative

Bone marrow cell morphology (January 29)

Bone marrow hyperplasia was active, with a normal myeloid/erythroid ratio, increased granulocyte proportion, thrombocytopenia, and no increase in blast cells or obvious pathological hematopoiesis observed.

Imaging results

Chest Computed Tomography (CT) (January 16)

**Figure 1:**
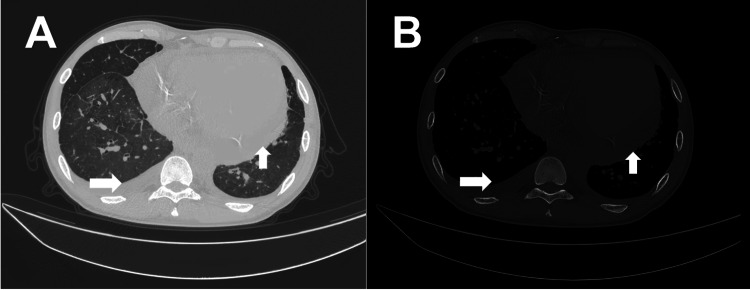
Multiserosal Effusions With Cardiopulmonary Abnormalities on Chest CT (A) Chest CT in lung window and (B) mediastinal window demonstrating pulmonary edema and pneumonia, bilateral pleural effusion, left heart enlargement, pericardial effusion, and ascites (representing multiple serous cavity effusions). CT: computed tomography

Chest CT revealed bilateral pulmonary edema combined with pneumonia, bilateral pleural effusion, left heart enlargement, pericardial effusion, and abdominal effusion (multiple serous cavity effusions) (Figure 1).

Cranial Magnetic Resonance Imaging (MRI) (January 17)

MRI showed multiple lacunar cerebral infarcts and ischemic foci in the bilateral corona radiata, bilateral frontal and parietal subcortical areas, and around the bilateral lateral ventricles, with mild brain atrophy, as well as softening foci in both cerebellar hemispheres.

Ultrasound (January 18)

Kidneys/ureters/bladder/prostate: The bilateral kidneys exhibited changes consistent with chronic renal insufficiency. There were cystic-occupying lesions in both kidneys, as well as prostate hyperplasia with calcification.

Thyroid/cervical lymph node: A hypoechoic nodule was seen at the lower pole of the left lobe of the thyroid (TR4). There was also a cystic nodule at the upper pole of the right lobe of the thyroid (T1), with no lymph node enlargement seen in the bilateral neck.

Hepatobiliary/pancreas: The liver has a normal size, with no occupying lesions seen, normal gallbladder size, thickened gallbladder wall, and a strong echogenic mass within the gallbladder, consistent with multiple gallstones. The spleen was enlarged, with strong echogenic foci, possibly indicating calcification, and multiple echogenic masses of undetermined nature. The pancreas is of normal size, with no occupying lesions seen.

**Figure 2:**
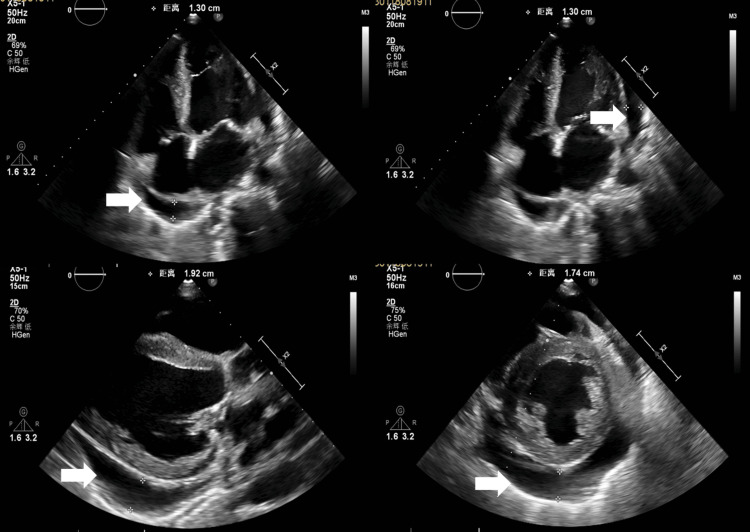
Echocardiographic Visualization of Moderate Pericardial Effusion Cardiac color Doppler ultrasound demonstrates a moderate pericardial effusion. The fluid accumulation is clearly visualized in the standard echocardiographic views, including the apical four-chamber view, parasternal long-axis view, and parasternal short-axis view.

Heart: The left heart was enlarged, with left ventricular wall thickening. The aortic and mitral valves were calcified. There was moderate mitral regurgitation, mild tricuspid regurgitation, reduced left ventricular ejection fraction, and moderate pericardial effusion (Figure 2).

Chest/Abdomen CT (February 6 and 10)

**Figure 3:**
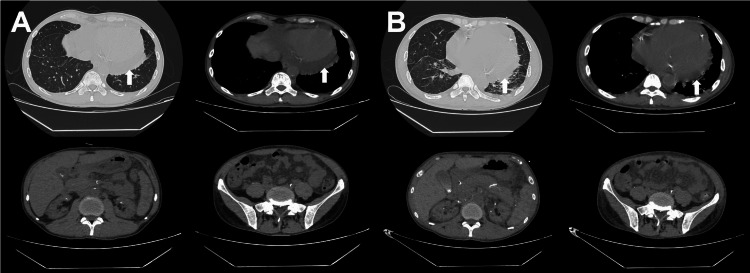
CT Scans of the Chest and Abdomen (A) CT obtained on February 6, 2023. (B) CT obtained on February 10, 2023. Lungs: Increased bilateral pulmonary edema and pneumonia. Pleural effusion on both sides. Heart: Left ventricular enlargement is now evident. Pericardial effusion remains stable. Abdomen/pelvis: Development of peritoneal and mesenteric edema. Small ascites (pelvic and peritoneal). Spleen: Multifocal hypodense lesions, compatible with splenic infarcts. CT: computed tomography

There was increased pulmonary edema and pneumonia in both lungs compared to previous scans, with small amounts of pleural effusion on both sides and enlargement of the left ventricle. The pericardial effusion was roughly similar. There was omental and mesenteric edema, and small amounts of fluid in the pelvic and abdominal cavities. Multiple slightly low-density lesions in the spleen were noted, suggesting splenic infarction (Figure 3).

Primary diagnosis

The primary diagnosis included systemic lupus erythematosus (active phase), acute renal failure (uremic phase), multi-organ dysfunction syndrome (kidneys, respiratory system, cardiovascular system, hematological system, and liver), pleural effusion in multiple cavities (thoracic cavity, pericardial cavity, and abdominal cavity), coronary artery disease involving three vessels, and secondary cerebral hemorrhage (thalamic hemorrhage extending into the ventricles).

Treatment

Initial Treatment

Initial treatment included regular hemodialysis, anti-infection therapy, blood pressure regulation, heart rate control, correction of hypoalbuminemia, correction of anemia, gastrointestinal conditioning, and nutritional support therapy after admission.

Complication Treatment

On January 17, the patient exhibited generalized seizures and symptoms of epilepsy, for which sodium valproate was administered as antiepileptic treatment. After treatment, on February 4, the patient began to experience fever, low blood pressure, persistent elevation of procalcitonin (PCT), abdominal distension, abdominal pain, and tenderness with rebound tenderness in the right abdomen. CT scans of the chest and entire abdomen on February 6 and February 10 indicated increased pulmonary edema and pneumonia in both lungs compared to previous scans, small amounts of pleural effusion on both sides, and enlargement of the left ventricle. The pericardial effusion was roughly similar. There was omental and mesenteric edema, and small amounts of fluid in the pelvic and abdominal cavities. Multiple slightly low-density lesions in the spleen were noted, suggesting splenic infarction. On February 15, an ultrasound-guided percutaneous cholecystostomy was performed. The patient exhibited multi-organ dysfunction (kidneys, hematological system, respiratory system, and cardiovascular system), with positive results for anti-double-stranded DNA antibodies (1:40), antinuclear antibodies (1:320, homogeneous type), and anti-nucleosome antibodies, indicating a possible autoimmune disease. Additionally, there was multi-serous cavity effusion (pleural, pericardial, and abdominal effusions) and thrombocytopenia (72×10⁹/L). A bone marrow biopsy was completed, ruling out primary hematological diseases, further supporting the systemic involvement characteristics of SLE.

SLE-Targeted Treatment

On February 17, a multidisciplinary team (MDT) consultation was conducted (involving nephrology, hematology and oncology, critical care, infectious diseases, gastrointestinal surgery, hepatobiliary and hernia surgery, radiology, cardiology, and pulmonology). SLE causing multisystem damage was considered, and lupus activity is currently suspected, indicating the need for anti-SLE treatment. However, due to the patient's infection not being fully controlled, his general condition is poor, and the critical nature of his illness poses risks for anti-SLE treatment, including exacerbating the infection and potentially leading to life-threatening spread. On February 19, the patient experienced chest tightness and abdominal discomfort, with significantly elevated high-sensitivity troponin levels, raising suspicion for coronary heart disease. The patient was transferred to cardiology and underwent coronary angiography on February 22, which indicated dominance of the right coronary artery, with lesions in all three major vessels. There was no significant stenosis in the left main artery, while the left anterior descending artery showed diffuse narrowing throughout, with chronic occlusion in the mid-segment and TIMI grade 0 antegrade flow; the circumflex artery exhibited diffuse narrowing, with severe stenosis of about 90% and TIMI grade 3 antegrade flow; the right coronary artery was diffusely calcified with a long segment of stenosis of about 90% in the distal segment and TIMI grade 3 antegrade flow. Because the family refused interventional treatment for the coronary arteries, postoperative management for coronary heart disease with three-vessel lesions was initiated, along with secondary prevention measures.

End-stage event

On February 25, the patient's condition worsened, experiencing shortness of breath and increased difficulty in breathing, low peripheral oxygen saturation under high-flow oxygen therapy, and low blood pressure. The patient could not undergo routine hemodialysis treatment due to hemodynamic instability and was transferred to the intensive care unit (ICU) for monitoring and treatment. Upon admission to the ICU, the patient exhibited seizures in the limbs, a left upper gaze, and persistent hypoglycemia despite high sugar maintenance, along with sustained high fever. Infection markers were elevated, renal and liver functions significantly deteriorated, blood cell counts were markedly reduced, and platelet levels were notably low. Immediate interventions included endotracheal intubation for mechanical ventilation, anti-infection treatment, and blood transfusions. On February 26, a sudden cerebral hemorrhage occurred, and the family opted to forgo invasive resuscitation efforts, leading to the patient's death.

## Discussion

Diagnosis and cause analysis of the patient

According to the 2019 European League Against Rheumatism/American College of Rheumatology (EULAR/ACR) classification criteria for SLE [7], the patient accumulated a total of 17 points (diagnostic threshold ≥ 10 points), specifically manifested as follows: in the immunological criteria, positive anti-double-stranded DNA antibodies (+6 points) and antinuclear antibody titer ≥ 1:80 (+3 points); in the clinical criteria, serositis (multiple serous cavity effusions, +6 points), thrombocytopenia (+2 points), and chronic renal insufficiency (with evidence of active kidney disease, +8 points). Although renal biopsy or complement testing was not performed, the patient's long history of hemodialysis and renal imaging changes (chronic lesions in both kidneys) suggested SLE-related kidney damage. Furthermore, despite seizures and cerebral hemorrhage not being included in the points, the possibility of neuropsychiatric lupus should still be considered. All the above findings meet the diagnostic criteria for SLE. The patient ultimately died from cerebral hemorrhage and multi-organ failure. The underlying pathophysiology can be attributed to persistent autoantibody-mediated endothelial injury and microthrombosis via immune complex deposition and complement activation, exacerbating pulmonary edema, pericardial effusion, and splenic infarction. Concurrently, thrombocytopenia and a hypercoagulable state associated with lupus anticoagulant likely contributed to the fatal cerebral hemorrhage, a known direct cause of mortality in SLE patients [8].

Moreover, the patient developed recurrent pulmonary infections, cholecystitis, and sepsis post-admission, creating a vicious cycle of "bidirectional deterioration" between infection and SLE activity. Uremia-induced immune dysfunction and long-term dialysis heightened infection susceptibility, which in turn amplified lupus flare-ups [9,10]. This interplay posed a therapeutic challenge: administering glucocorticoids or immunosuppressants in the setting of uncontrolled sepsis risked exacerbating infectious spread.

The treatment dilemma lies in the fact that glucocorticoids or immunosuppressants may accelerate the spread of infection in uncontrolled septic states. The collapse of the cardiovascular system further worsened the condition, with triple-vessel coronary artery disease, pericardial effusion, and reduced left ventricular ejection fraction triggering hemodynamic collapse during septic shock, while hypoalbuminemia exacerbated tissue edema, leading to heart and lung function decompensation [11-13]. Metabolic disturbances manifested as refractory hypoglycemia, possibly resulting from sepsis-related glucose utilization abnormalities, liver failure-induced gluconeogenesis impairment, and chronic malnutrition [14]. Persistent seizures and intracranial hemorrhage reflected the combined effects of blood-brain barrier disruption and uremic toxin accumulation on the central nervous system.

This case underscores that renal insufficiency can be the sole initial manifestation of SLE, necessitating early immunological screening even in the absence of classic features. It is crucial to initiate individualized immunomodulatory treatment promptly, ideally before the condition progresses to sepsis or once the infection is controlled. The overlap between complications of CKD and manifestations of SLE creates diagnostic blind spots. Key clues such as thrombocytopenia, hypoalbuminemia, and polyserositis were initially misattributed to uremic or septic complications. The low-titer anti-dsDNA (1:40) and ANA positivity (1:320, homogeneous pattern) were critical, albeit subtle, immunological indicators [15]. In men with unexplained multi-organ dysfunction, even weakly positive autoantibody results should prompt thorough evaluation for SLE to avoid delayed diagnosis and worsened outcomes.

Rarity and diagnostic challenges in male patients with SLE

SLE exhibits significant gender disparity, with men representing only 10%-20% of cases, often presenting with more severe renal and hematological involvement [1-3,6]. This case illustrates an exceptionally rare scenario: a male patient with isolated CKD as the initial SLE manifestation, masking the autoimmune etiology for five years. Literature indicates that <5% of SLE cases present with significant renal impairment devoid of classic features such as malar rash or arthritis, frequently leading to misdiagnosis as primary CKD or hypertensive nephropathy [4]. The prolonged focus on renal replacement therapy in this patient diverted attention from underlying autoimmune pathology, delaying SLE recognition until multisystem failure ensued. Such diagnostic delays are clinically consequential, correlating with accelerated progression to end-stage renal disease (ESRD) and higher mortality [5,16].

Imperative of multidisciplinary collaboration

The patient's rapid decline into multi-organ dysfunction syndrome underscores the vital role of multidisciplinary team (MDT) collaboration in managing complex SLE cases. Initial consultations between nephrology and rheumatology services facilitated prompt autoimmune serology testing, confirming SLE despite atypical presentation. This collaboration not only provided a basis for the etiological diagnosis but also laid the foundation for subsequent treatment plans. As the patient deteriorated, the involvement of intensive care, infectious disease, cardiology, and other specialties enabled a coordinated approach to treating septic shock, coronary artery disease, and coagulation disorders, thereby securing precious treatment time for the patient. However, the MDT faced a dilemma: initiating immunosuppressive therapy during the active infection phase could worsen sepsis, while delaying treatment risked lupus progression. This underscores the need for ongoing MDT collaboration to perform a dynamic risk-benefit assessment. Unfortunately, despite the multidisciplinary team's best efforts, the patient ultimately succumbed to severe illness. This case highlights that early and comprehensive multidisciplinary involvement is essential for developing personalized strategies that balance immunosuppression and infection control, thereby optimizing treatment outcomes for high-risk SLE patients.

Lessons for clinical practice

First, maintaining a high index of suspicion for SLE in atypical patient populations is paramount. This case involved a 37-year-old man with chronic renal failure lacking classic lupus features. For patients with CKD who develop unexplained multi-organ dysfunction, especially when accompanied by hematological abnormalities (such as pancytopenia), multiple serous cavity effusions, and positive autoimmune serology, the possibility of lupus nephritis should be considered. Early recognition of these atypical symptoms is crucial for improving patient outcomes.

Second, early integration of immunological screening is essential. The diagnosis in this patient was delayed due to the absence of typical symptom manifestations, likely resulting in irreversible organ damage. Early comprehensive rheumatological screening and prompt initiation of treatment for systemic lupus erythematosus based on evolving clinical presentations may prevent progression to irreversible multi-organ dysfunction. For high-risk groups, prioritizing rheumatological examinations facilitates timely interventions before severe complications arise, thereby maximizing the treatment window.

Third, implementing MDT collaboration early is vital for comprehensive management. Early involvement in MDT management allows for a comprehensive assessment of infection risk, organ function, and disease activity, enabling the development of personalized treatment plans. At the same time, continuous monitoring and adaptive strategies are essential to address the complex interactions between infection and autoimmunity and to optimize treatment outcomes.

## Conclusions

This case highlights the diagnostic and therapeutic challenges of SLE in male patients presenting with CKD as the initial manifestation. The atypical presentation, characterized by isolated renal insufficiency preceding sudden multi-organ failure, underscores the necessity of maintaining a high clinical suspicion for SLE in men with unexplained multi-organ dysfunction, even in the absence of classic lupus features, and prioritizing early immunological screening to prevent diagnostic delays. Multidisciplinary collaboration is essential to balance immunosuppression with infection control and address organ-specific complications, while therapeutic dilemmas arising from the bidirectional relationship between infection and SLE activity necessitate individualized strategies. Despite these efforts, the patient's rapid decline and fatal outcome emphasize the aggressive nature of SLE-related multi-organ dysfunction in uremic patients, particularly when exacerbated by infections and cardiovascular complications. The case also highlights the poor prognosis associated with delayed SLE diagnosis in CKD patients, reinforcing the importance of early intervention and tailored management to improve survival and quality of life. Future efforts should focus on raising awareness of atypical SLE presentations in men, optimizing multidisciplinary care models, and exploring novel therapies for high-risk populations.
